# A scoping review of palliative care for children in low- and middle-income countries

**DOI:** 10.1186/s12904-017-0242-8

**Published:** 2017-11-25

**Authors:** Hatoko Sasaki, Marie-Charlotte Bouesseau, Joan Marston, Rintaro Mori

**Affiliations:** 1 Department of Health Policy, National Center for Child Health and Development, 2-10-1 Okura, Setagaya, Tokyo, 157-8535 Japan; 2 Service Delivery and Safety, World Health Organization, 20 Avenue Appia, 1211, 27 Geneva, Switzerland; 3 International Children’s Palliative Care Network, 2 Langenhoven Street, Dan Pienaar, Bloemfontein, 9301 South Africa

**Keywords:** Palliative care, Scoping review, Children, Low- and middle-income countries

## Abstract

**Background:**

Ninety-eight percent of children needing palliative care live in low- and middle-income countries (LMICs), and almost half of them live in Africa. In contrast to the abundance of data on populations in high income countries, the current data on populations in LMICs is woefully inadequate. This study aims to identify and summarize the published literature on the need, accessibility, quality, and models for palliative care for children in LMICs.

**Methods:**

A scoping review was performed following the method of Arksey and O’Malley. Systematic searches were conducted on PubMed and Google Scholar using the main keywords, ‘children AND palliative care OR terminal care OR hospice OR end of life AND developing countries OR LMICs.’ Additional publications were obtained by handsearching. Papers were only included if they reported on the need, accessibility, quality, and models for palliative care for children in LMICs.

**Results:**

Fifteen papers met the inclusion criteria for review. Of these, 10 assessed need, seven examined availability and/or accessibility, one assessed quality, and one examined the models. We found an urgent need for palliative care, particularly in the training for health workers and improving poor availability and/or accessibility to palliative care in terms of factors such as medication and bereavement support. The best practice models demonstrated feasibility and sustainability through cooperation with governments and community organizations. The quality of pain management and emotional support was lower in LMICs compared to HICs.

**Conclusion:**

Although we found limited evidence in this review, we identified common challenges such as the need for further training for health workers and greater availability of opioid analgesics. While efforts to change the current systems and laws applying to children in LMICs are important, we should also tackle underlying factors including the need to raise awareness about palliative care in public health and improve the accuracy of data collection.

**Electronic supplementary material:**

The online version of this article (10.1186/s12904-017-0242-8) contains supplementary material, which is available to authorized users.

## Background

Every year around the world, an estimated 20 million people require palliative care if the symptoms they experienced at the end of their life are seen as an indicators of their need. Of these individuals, 78% live in low- and middle-income countries (LMICs)^1^ [1–3]. Ninety-eight percent of children requiring palliative care live in LMICs, and approximately half live in Africa [2, 3]. Children may have a high incidence of congenital anomalies and genetic conditions, and mortality is highest in the neonatal period [4]. The World Health Organization (WHO) acknowledged the importance of palliative care, especially in LMIC populations, by passing its resolution to strengthen palliative care at the 2014 World Health Assembly (WHA) [5]. Special mention of palliative care for children was made in the WHA Resolution.

The WHO describes palliative care as a way of enhancing the quality of life of patients and their families as they experience the challenges of serious illness by preventing and reducing pain and suffering through early identification of disease, accurately assessing and managing pain, and addressing physical, psychosocial, and spiritual issues [6]. Palliative care for children is a special, yet closely related field to adult palliative care and aims to provide care for the body, mind, and spirit of a child and support to the family, and to be available from the time a life-threatening condition is diagnosed. Communication with younger children tends to be more difficult than with adults and changes as the child matures [7]. Assessment of pain level [8] and quality of life [9, 10] of pediatric patients is challenging due to variations in children’s verbal competence and reflective ability depending on their developmental stage. It is also hindered by the fact that a large number of children with life-threatening or life-limiting illnesses also suffer from intellectual and developmental disabilities [11] and may therefore not be able to communicate well. Moreover, illness trajectories vary greatly even if children receive the same diagnosis [12]. Pharmacokinetics and prognostication are different in pediatric patients than in adults, and therefore, psychotherapeutic and other treatment approaches need to be modified. Ethical considerations are also important, as in many cases children lack decision-making capacity, and conceptual agreement between the child, family, and health professionals is required [13]. Particularly, withholding and withdrawing life-sustaining treatments are common dilemmas in children’s palliative care (CPC) [7]. The guidelines of the Royal College of Paediatrics and Child Health describe four aspects of child involvement in decision-making that every health professional should be aware of: the child should 1) be informed, 2) be consulted, 3) have his or her views taken into account in decision-making, and 4) be respected as the main decision-maker [14].

Given that 98% of children needing palliative care live in LMICs [3], the WHO has placed great emphasis on implementing the WHA resolution in those countries. Implementation focuses on revising laws and procedures to improve access to opioid pain relief, including training health workers in palliative care and providing palliative care services through primary health care centers and in the home [5]. Regardless of the high percentage of children needing palliative care, there is insufficient evidence on the current health conditions of populations in LMICs compared to those of populations in high-income countries (HICs).The literature indicates that the grave absence of CPC services in LMICs are due to financial issues, shortage of trained staff, and unavailability of medical opioids [15, 16]. On the other hand, despite the expansion of CPC services in HICs, many children with incurable diseases still do not receive palliative care [17, 18]. The barriers to optimal palliative care include long-term funding for essential medicines, treatment, lack of training for health professionals, lack of human resources [4], the small number of referrals to palliative care [19], inadequate training and expertise [20], inadequate pain assessment and management [21], and ethical issues [22]. Some of the same problems are also encountered in LMICs.

A systematic review of the reported availability and gaps in pediatric palliative care in LMICs was published in 2014 [23]. The objective of the review was to assess how the core elements of pediatric palliative care were being provided. However, no recent, comprehensive review of pediatric palliative care in LMICs addressing broad areas of evidence has been published. The purpose of the present review was to identify and summarize the current needs, accessibility, quality, and models of palliative care for children in LMICs.

## Methods

A scoping review of the literature was conducted. Scoping reviews aim to map the key concepts underlying a research area. This approach is particularly appropriate when the main sources and types of available evidence are complex or have not been reviewed comprehensively before [24]. For this review, we adapted the 6-step methodological framework outlined by Arksey and O’Malley [24] consisting of 1) identification of the research question; 2) identification of relevant studies; 3) selection of studies for review; 4) charting the data; 5) collating and summarizing the results, and 6) consulting (optional). As per the method of Arksey and O’Malley [24], the quality appraisal of the studies was beyond the scope of this review.

### Step 1: Identification of research question

The primary objective of this review was to identify and summarize the current needs, accessibility, quality, and models of palliative care for children in LMICs. Before beginning the review we identified the research question based on the fact that the available evidence on children’s palliative care in LMICs has not yet been broadly captured and summarized.

### Step 2: Identification of relevant studies

To locate relevant, recent studies, we performed a systematic search of reports published between February 1, 2006 and February 1, 2016 using PubMed and Google Scholar. Hand and reference list searching was also conducted to identify additional relevant publications of organizations such as the Worldwide Hospice Palliative Care Alliance (WHPCA) and the International Children’s Palliative Care Network (ICPCN). A detailed description of our search strategy is presented in Additional file 1.

### Step 3: Selection of studies for review

After duplicate elimination, titles and/or abstracts were screened, and studies relevant to the research questions were identified. Full texts of the retained studies were read and selected based on the following criteria:

#### Inclusion criteria

- Studies on the need, accessibility, quality, and models of palliative care for children in low- and middle-income countries according to per capita gross national income (GNI) of $1025 or less in 2015 [1];
- Studies published in the English language.

#### Exclusion criteria

- Studies on the need, accessibility, quality, and models of palliative care for children in high-income countries;
- Studies on the need, accessibility, quality, and models of palliative care for adults;
- Protocol papers.

### Step 4: Mapping the data

Information from the selected studies was sorted and organized into the following categories: authors, year of publication, country of the leading author, objective, country of the study, population, key findings, and theme (Table 1).

**Table 1:** Summary of reviewed studies

Authors/year of publication	Country of the leading author	Aim	Country/region of the study	Population	Design, Methodology	Key findings	Theme
Amery J (2009) [7]	UK	Book chapter about communicating with children and their families	Africa	N/A: Book chapter	Book chapter	Communicating with children in pediatric palliative care is one of the greatest education needs of health workers in this field.	Needs
Amery JM, et al. (2009) [36]	UK	To evaluate children’s palliative care service designed specifically for a resource-poor sub-Saharan African setting.	Sub-Saharan Africa	Children (*N* = 11), children’s parents/legal caregivers (*n* = 12); and all hospice and hospital staff on the cancer ward (*n* = 10).	Quantitative retrospective, comparative survey and cross-sectional, non-interventional interview survey.	There were increases in referrals, the number of children in programs, morphine and chemotherapy prescriptions, and improved compliance for a cost of $100 per child. Key strengths of the service were free drugs, food, play, learning, and staff attitude. Weaknesses were insufficiency of the above strengths, poor attitude of hospital staff, a lack of school fees and poor treatment compliance rates. Suggestions included more of the strengths and greater accessibility service locations.	Availability/Needs
Caruso Brown AE, et al. (2014) [23]	US	To review data on palliative care services available to young people with life-limiting conditions in LMICs and assess core elements of availability, gaps, and underreported aspects.	21 countries (8 in Europe and Central Asia, 7 in the Middle East and North Africa, 3 in Sub-Saharan Africa, 2 in Latin America and the Caribbean, 1 in South Asia)	34 studies included in the systematic review.	Systematic review	The most significant gaps were found in national health system support (unavailable in 7 of 17 countries with programs reporting), specialized education (unavailable in 7 of 19 countries with programs reporting), and comprehensive opioid access (unavailable in 14 of 21 countries with programs reporting).	Accessibility
Cardenas-Turanzas M, et al. (2015) [32]	US	To assess the need for palliative and end-of-life care for children dying in public hospitals in Mexico.	Mexico	2715 children aged 1–17 years; mean age: 8 years	Retrospective analysis of mortality data	A large number of deaths were related to complex chronic conditions, highlighting the need for adequate funding of professional education and palliative care initiatives for children.	Needs
Collins K and Harding R (2007) [30]	UK	To measure the prevalence of multidimensional palliative care needs of patients with HIV in Muheza, Tanzania.	Tanzania	82 children aged under 16 years	Data from a prospective 1-month patient census.	The young population, particularly the 82 patients under 16, is in great need of specialist pediatric palliative care skills relevant to HIV, including methods for assessing children’s needs and clinical skills and formulations for symptom control among children.	Needs
Connor SR, et al. (2014) [37]	UNICEF/ICPCN	To analyze existing secondary data to estimate the palliative care needs among children and to explore with service providers the key gaps in response.	South Africa, Kenya and Zimbabwe	All infants, children and adolescents having one or more conditions listed in the inclusion criteria as agreed by the WHO.	Cross-sectional mixed methods-study which uses an analysis of prevalence and mortality data; literature review; interviews; surveys.	The need for palliative care for children is recognized in all countries. There are two categories in CPC: general (should be provided by any primary health care system) and more specialized (additional services needed).	Needs
Delgado E, et al. (2010) [31]	US	To assess the availability and quality of palliative care for children with cancer according to national per capita income.	LIC	Clinicians who care for children with catastrophicillnesses worldwide (*N* = 262).	Questionnaire (pediatric oncology practice variables, availability of palliative care services, quality of palliative care services)	High potency opioids and adjuvant drugs were significantly less available in LICs. Physicians in LICs were significantly less likely than those in HICs to report high-quality pain control, non-pain symptom control, and emotional support; bereavement support; interdisciplinary care; and parental participation in decisions.	Availability/Quality
Downing J, et al. (2012) [26]	UK	To examine the development of CPC in different regions of the world: Argentina, New Zealand, Romania, Uganda, and the United Kingdom.	Global	N/A: Commentary	Commentary	The ‘Charter of rights for life-limited and life-threatened children’ sets out an international standard of support for all children living with life-limiting or life-threatening conditions and their families and asserts that providing CPC is not just morally correct but also a basic human right; and that a lack of availability of such services for so many children is not acceptable.	Needs/Availability
Downing J, et al. (2016) [25]	UK	To review the status of CPC services in LMICs by providing examples of the best practices among service models and to review published research.	LMICs focusing on Malawi, Indonesia, Belarus	N/A: Literature review	Narrative literature review	Palliative care for adults has generally received more attention compared to care for children and young people, indicating greater attention and support are needed in education, clinical practice, funding and research. .	Model
Lan KG & Yun LW (2015) [34]	Malaysia	To examine parents’ experiences of the end of life care of their children, and to collect their views, needs, and concerns regarding the level of support.	Malaysia	Children aged 2–14 years	Focus groups and in-depth interviews based on semi-structured interview protocol.	Common themes were strengths and weaknesses of the healthcare system and procedures in palliative care, such as symptom control, closed communication, and lack of support and anticipatory guidance prior to their child’s death.	Needs
Hessissen L & Madani A (2012) [27]	Morocco	To describe the achievements and challenges of pediatric oncology in Morocco.	Morocco	N/A: Supplement	Supplement	In spite of recent improvements, pediatric oncology in Morocco still needs to achieve better performance, specifically, improving survival rate, reducing treatment abandonment, developing new techniques, improving quality of life, and creating data collection teams.	Needs/Availability
Nakawesi J, et al. (2014) [33]	Uganda	To report on the palliative care needs of HIV-exposed and HIV-positive children admitted to the inpatient pediatric unit.	Uganda	243 children aged 0–18 years	Retrospective observational study	Psychological needs were identified as: antiretroviral treatment (ART) counseling (36%), HIV counseling and testing for the child and family (18%), adherence support (15%), and others (3%). Spiritual needs were identified as: ceasing ART due to spiritual beliefs (81%), loss of hope due to severity of illness (5%), and others (14%).	Needs
Shad A, Ashraf MS, Hafeez H (2011) [28]	Pakistan	To describe the development of palliative care services in Pakistan.	Pakistan	N/A: Supplement	Supplement	Pediatric palliative care is in the early stages of development. Two children’s hospitals created small inpatient palliative care units. However, lack of trained staff, lack of knowledge in palliative care, inadequate supply of morphine, and no outreach were recognized as major problems.	Needs/Availability
Silbermann M, et al. (2012) [29]	Lebanon	To describe accomplishments and challenges of palliative cancer care including accessibility and availability of medications and training for caregivers for adult and pediatric cancer patients.	Middle-eastern countries	N/A: Supplement	Supplement	There continue to be large disparities in morphine and opioid consumption, and these drugs continue to be unavailable for medical purposes. Hundreds of professionals took part in educational and training activities over the past 6 years, and became the core of the trained caregivers who initiated reforms in their respective countries.	Needs/ Availability
Weaver MS, et al. (2015) [35]	US	To investigate the pediatric and palliative care elements in cancer control plans.	Africa	Children aged 0–14 years	Comparative content analysis of accessible national cancer control plans in Africa.	Eleven national plans identified palliative care needs representing 24% of the estimated Africa-wide burden for children aged 0–14 years. Four national plans specified pediatric palliative needs.	Needs/ Availability

### Step 5: Collating and summarizing the results

In the process of collating and summarizing the findings, the extracted evidence was repeatedly reviewed. Results were summarized to present an overview of the current evidence on children’s palliative care by theme. We performed content analysis of the themes to identify further contextual factors (e.g. educational needs of health professionals, need for emotional support, accessibility and/or availability of medication, etc.).

## Results

### Description of identified studies

**Fig. 1:**
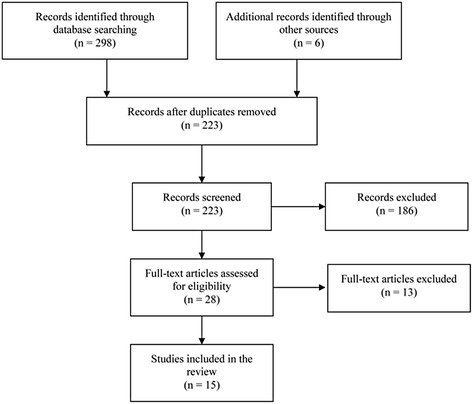
Flow chart of the review procedure

Fifteen potentially eligible papers were identified after excluding duplicates and judging the relevance of the studies (Fig. 1). Table 1 summarizes the characteristics of the studies included in the review. The types of study varied: one was a systematic review [23], one was a narrative literature review [25], four were supplements or commentaries [26–29], four were quantitative studies [30–33], two were qualitative studies [34, 35], two were mixed-method studies [36, 37], and one was a book chapter [7]. Of these, 12 studies evaluated needs [7, 26, 28–30, 32–34, 36, 37], eight studies examined availability and/or accessibility [23, 26–29, 31, 35, 36], one study assessed quality [31], and one study addressed the model [25]. Seven studies mentioned more than one theme. Geographical distribution among the studies was examined. Five studies covered a single country including Malaysia [17], Mexico [6], Morocco [12], Pakistan [13] and Uganda [10]. Nine studies covered multiple countries or specific regions [7–9, 14–16, 18–20].

### Needs of palliative care

#### Need estimation based on disease burden

Two studies estimated the need for children’s palliative care in Africa. In the first study the rate of need was reported as 3.75 times higher in Kenya, 4.75 times higher in South Africa, and 5.6 times higher in Zimbabwe than in the United Kingdom [37]. Compared to the estimated need in these three countries, the number of children receiving palliative care was considerably low. The second study reviewed 11 national plans addressing palliative care needs, which accounted for 24% of the total continental burden for children aged 0 to 14 years in Africa using World Bank population data [38] and burden estimates [2]. Of these plans, four identified pediatric palliative needs, and three described the importance of training general healthcare workers in pediatric cancer management [35].

#### Educational needs of health professionals

Education of health professionals was the most frequently mentioned issue in studies that reported on palliative care needs [7, 26, 29, 32, 36]. Due to the lack of training, health workers reported having difficulty caring for and communicating with their pediatric patients [7, 36]. Additionally, healthcare workers were reluctant to use opioid medications due to concerns that the drugs might lead to dependence syndrome or respiratory distress in patients [29]. Further health education was found not only to be necessary for healthcare providers but also for parents, caregivers, and guardians [32] given the lack of awareness evident in their attitudes about palliative care services and how to communicate with healthcare providers.

#### Emotional and psychosocial support needs

The need for emotional and psychosocial support was also addressed by three studies [33, 34, 37]. Healthcare workers in Zimbabwe provided psychosocial support and antiretroviral therapy (ART) to children born with and living with HIV, many of whom were abandoned [29]. Of 243 children admitted to the inpatient pediatric ward in Uganda, 21% showed a need for social support related to issues such as financial instability and child neglect, and 18% to 36% presented with more than one psychological need including ART counseling, HIV counseling and testing for the child and family, and adherence support [29]. Parents in Malaysia reported a lack of support and anticipatory guidance from healthcare providers in the end-of-life care (EoLC) for their children [34].

### Accessibility and/or availability of palliative care

#### Medication

Three studies reported on the poor availability and accessibility of medication [26, 27, 29]. A large disparity in the availability of opioids among Middle Eastern countries was noted [29]. In the Middle East, injectable morphine was available only in hospitals in Turkey, Israel, and Iran [29]. Although morphine or opioid therapy was available in Morocco, it was not always adapted to pediatric patients due to the country’s strict laws [27]. In addition to availability, the cost of medications was also a major obstacle to palliative care in Argentina [26].

#### Services and programs

A quantitative, retrospective, comparative survey forming part of a mixed quantitative- qualitative study on a children’s palliative care service in sub-Saharan African found an increase in the number of referrals from 2006 to 2007, children in programs, morphine and chemotherapy prescriptions, and compliance with treatment costs compared to the first survey period. However, the staff had significant concerns about the accessibility of services and abandonment of treatment by patients or their families [29].

One study surveying physicians who cared for children with cancer examined the availability and quality of palliative care in low-income countries (LICs). Comfort care medications (24.3%), palliative care (21.9%), and oncology (14.8%) out-of-pocket payments were most likely to be needed in LICs [31]. Availability of specialized palliative care, pain management, bereavement support, and institutional or nationwide decision-making support was inversely related to the level of income [29]. High potency opioids and pharmaceutical aids were significantly less available in LICs [31].

A systematic review of the reported availability and gaps in pediatric palliative care found that the most common challenges were presented by the unavailability of support services from the national health system (unavailable in seven of 17 LMICs with programs reporting), specialized education (unavailable in seven of 19 LMICs with programs reporting), and broad opioid access (unavailable in 14 of 21 LMICs with programs reporting) [23].

### Models of palliative care

Innovative palliative care programs making use of available resources and adapted to situations in a specific country or region have been developed in a number of LMICs. Downing et al. [25] provided three examples of the best practice models in Malawi, Indonesia, and Belarus. Umodzi is a CPC program based at the Queen Elizabeth Central Hospital in Malawi that has been integrated into the pediatric and general health system since its inception in 2001. Yayasan Rumah Rachel in Indonesia provides home-based care for children with HIV or cancer and their families through a network of care providers. Capacity building and policy advocacy are important aspects of the service, which also provides training and mentoring for medical professionals and allied health workers, and supports the development of a national palliative care policy. The Belarusian Children’s Hospice (BCH), originally established to care for children after the Chernobyl nuclear disaster, promotes advocacy, education, and mentorship and has been very influential in the development of CPC in Belarus and neighboring countries. The inpatient services in these countries tend to be limited; however, the core service of providing CPC at home is implemented by teams of doctors, nurses and carers. BCH also runs two social and psychological care programs for children and young adults. As a result of working closely with the national government, legislation has been passed to enable all families with children in palliative care to receive 2 weeks’ free respite care per year. Strong leadership and effective collaboration with the government and community-based organizations are the key to the success of these models.

### Quality of palliative care

Only one study reported on the quality of palliative care [31]. Physicians in LICs tended to disagree with quality statements more often than their colleagues in high-income countries (HICs). Only 57% of physicians in LICs agreed with the quality statements on effective pain management in comparison to 87% in HICs. The remaining quality comparisons, including effective management of non-pain symptoms, good emotional support, good bereavement care, interdisciplinary care, and parental participation in care decisions, showed a lower quality for LICs [31].

## Discussion

Our review of 15 studies on palliative care for children covered multiple LMICs in Africa, Asia, the Middle East, and Latin America. The reviewed studies all stated an urgent need for children’s palliative care including training for health providers and emotional/psychosocial support for patients and their families. The studies also noted poorer accessibility and availability of palliative care including medication and bereavement support. The best practice models showed that strong leadership and effective collaboration with the local government and organizations were essential for success. The quality of palliative care including pain management and emotional support was poorer in LICs than in HICs. When we organized the current literature by theme, it became clear that these themes were not independent of one another. Although palliative care in LMICs has been improving [27, 29, 39], children did not always benefit from these developments [23, 37]. Despite the differences in social background and economic level within the LMIC populations, our findings suggested that these populations shared certain issues and barriers.

Most studies on the need for palliative care emphasized the importance of improving health education. Three levels of educational need were identified: 1) basic training in palliative care for all healthcare professionals; 2) intermediate training for those regularly working with patients with life-threatening conditions, and 3) advanced training for managing patients with more complex symptoms [2]. When considering methods of education in Africa, there are several challenges to keep in mind, including the availability of educators, the effect on employers when staff take time off for training, travel distances, administration, and cost [40]. Distance learning may be useful for countries facing these challenges. ICPCN has developed online training in children’s palliative care [41]. The aims of the initial training courses were understanding the concept of pediatric palliative care, assessing and managing the pain and psychological problems experienced by pediatric patients, communicating and playing with children, and understanding grief, bereavement, dying, and death [41]. The online training course seemed to be effective, judging from more than 70% of participants’ reporting positive changes in their knowledge, skills, and attitudes towards CPC after taking the course [41]. Such training may improve healthcare workers’ confidence and competence in providing palliative care. Of the physicians who used Cure4Kids, a free online education program for CPC, 72.9% indicated that they were competent to provide care. These responses did not differ significantly by economic level [31].

The use of opioid analgesics in pediatric palliative care has more than doubled globally from 2001 to 2003 and 2011 to 2013, but remains low in Africa, Asia, Central America, the Caribbean, South America, and eastern and southeastern Europe [42]. This implies that the use of opioids has increased in only certain high-income countries. Restricted access to opioid analgesics in a large number of LMICs is a barrier that still needs to be overcome. It is not surprising that this review identified only a small number of studies on the quality and models of CPC because assessing the quality of palliative care and establishing good models fall outside the purview of basic palliative care services. While it is important to make every effort to change laws, train health workers, and provide basic palliative care services, it is also crucial to tackle fundamental factors underlying these challenges and manage available resources efficiently.

First, health education and awareness about palliative care among the general population need to be increased. Many people still tend to think that palliative care equals EoLC, which includes palliative care [43].Those living in a country where communicable diseases are the main cause of death may believe that palliative care is a luxury available only in HICs. Whether in an LIC or HIC, good pediatric palliative care is essential in an acute inpatient setting judging by the large body of evidence showing the need for EoLC during short-term hospitalization [44]. Advanced care or hospice care is one part of palliative care. When considering palliative care as holistic care as defined by the WHO [6], anyone with a severe and incurable illness can benefit from a palliative care approach and services, regardless of the stage of disease. Second, one of the major issues underlying research in LMICs is that the reported data are prone to underestimating the need for palliative care. Accurate data collection is essential to developing sound research and practices. Finally, home-based care is a key facet of palliative care especially in LMICs. The best practice models of palliative care in this review demonstrated that CPC is feasible and sustainable if it is done in cooperation with the government and community organizations. Other countries or regions can maximize their resources and develop their own models by learning from the strengths of the reported instances.

This review may have a number of limitations. First, the number of articles found and included in the analysis may have been limited due to searching only in PubMed and Google Scholar. Second, this review only included studies published in English. There may be relevant publications that are available only in languages other than English. Third, although we used a number of free-text and MeSH terms addressing the needs, accessibility, quality, and models of palliative care for children in LMICs, there is a possibility that non-reviewed studies may have used other terms with a similar meaning. Finally, due to the small number of reviewed studies, our findings may not describe the whole scope of our current knowledge of pediatric palliative care in LMICs.

## Conclusions

Our findings demonstrated an urgent need for palliative care in LMICs, particularly with respect to training for health workers and improving accessibility/availability of palliative care including medication and bereavement support. The best practice models showed that strong leadership and effective collaboration with the local government and organizations are essential for success. The quality of pain management and emotional support was poorer in LICs than in HICs. Although this review identified a limited number of studies on palliative care for children in LMICs, the challenges, such as training for health workers and availability of opioid analgesics, were common to different LMIC settings. Resource investment should focus on these challenges as well as foundational factors including recognition of palliative care in public health and accurate data collection in a framework of national policies and plans. Further research is clearly required to develop a body of evidence that is adequate to support effective service and policy development.

## Additional file

Additional file 1: The discription of the search strategy. (DOCX 20 kb)

## Abbreviations

ART: Antiretroviral therapy
BCH: Belarusian Children’s Hospice
CPC: Children’s palliative care
EoLC: End-of-life care
GNI: Gross national income
HICs: High-income countries
ICPCN: International Children’s Palliative Care Network
LMICs: Low- and middle-income countries
WHA: World Health Assembly
WHO: World Health Organization
WHPCA: Worldwide Hospice Palliative Care Alliance

## Footnotes

1 LMICs are defined as those with a per capita gross national income (GIN) of $1025 or less based on the World Bank’s analytical income classification of economies in 2015.

## References

[1] The World Bank. New country classifications by income level. https://blogs.worldbank.org/opendata/new-country-classifications-2016. Accessed 25 March 2017.

[2] Alliance, Worldwide Palliative Care, and World Health Organization. "Global atlas of palliative care at the end of life." London: Worldwide Palliative Care Alliance. 2014.

[3] World Health Organization. Global Health Estimates. Causes of Death 2000-2011; 2013.

[4] World Health Organization. "Planning and implementing palliative care services: a guide for programme managers." 2016.

[5] World Health Organization. Strengthening of palliative care as a component of comprehensive care throughout the life course. 2014. http://apps.who.int/gb/ebwha/pdf_files/WHA67/A67_R19-en.pdf?ua=1. Accessed 14 Sept 2016.

[6] World Health Organization. WHO Definition of Palliative Care. http://www.who.int/cancer/palliative/definition/en/. Accessed 14 Sept 2016.

[7] Amery J (2009). Children's palliative care in Africa.

[8] Committee on Psychosocial Aspects of Child and Family Health (2001). The assessment and management of acute pain in infants, children, and adolescents. Pediatrics.

[9] Drotar D: Measuring health-related quality of life in children and adolescents: implications for research and practice: Psychology Press, Hove, United Kingdom; 2014.

[10] Chaplin JE, Koopman HM, Schmidt S (2008). DISABKIDS smiley questionnaire: the TAKE 6 assisted health-related quality of life measure for 4 to 7-year-olds. Clin. Psychol. Psychother..

[11] Novak I, Hines M, Goldsmith S, Barclay R: Clinical prognostic messages from a systematic review on cerebral palsy. Pediatrics 2012; 130: pp. e1285-e1312 10.1542/peds.2012-0924 23045562

[12] Klick JC, Ballantine A (2007). Providing care in chronic disease: the ever-changing balance of integrating palliative and restorative medicine. Pediatr Clin N Am.

[13] Michelson KN, Steinhorn DM (2007). Pediatric end-of-life issues and palliative care. Clin Pediatr Emerg Med..

[14] Children’s Palliative Care Guidelines. Royal College of Paediatrics and Child Health, London 2011.

[15] Harding R, Higginson IJ (2005). Palliative care in sub-Saharan Africa. Lancet.

[16] Knapp C, Woodworth L, Wright M, Downing J, Drake R, Fowler-Kerry S, Hain R, Marston J (2011). Pediatric palliative care provision around the world: a systematic review. Pediatr Blood Cancer.

[17] Behrman RE, Field MJ: When children die: improving palliative and end-of-life care for children and their families: National Academies Press, Washington DC; 2003. 25057608

[18] Widger K, Davies D, Drouin DJ, Beaune L, Daoust L, Farran RP, Humbert N, Nalewajek F, Rattray M, Rugg M (2007). Pediatric patients receiving palliative care in Canada: results of a multicenter review. Arch. Pediatr. Adolesc. Med..

[19] Fowler K, Poehling K, Billheimer D, Hamilton R, Wu H, Mulder J, Frangoul H (2006). Hospice referral practices for children with cancer: a survey of pediatric oncologists. J Clin Oncol.

[20] Davies B, Sehring SA, Partridge JC, Cooper BA, Hughes A, Philp JC, Amidi-Nouri A, Kramer RF (2008). Barriers to palliative care for children: perceptions of pediatric health care providers. Pediatrics.

[21] Wolfe J, Grier HE, Klar N, Levin SB, Ellenbogen JM, Salem-Schatz S, Emanuel EJ, Weeks JC (2000). Symptoms and suffering at the end of life in children with cancer. N Engl J Med.

[22] Kunin H (1997). Ethical issues in pediatric life-threatening illness: dilemmas of consent, assent, and communication. Ethics Behavior.

[23] Caruso Brown AE, Howard SC, Baker JN, Ribeiro RC, Lam CG (2014). Reported availability and gaps of pediatric palliative care in low-and middle-income countries: a systematic review of published data. J Palliat Med.

[24] Arksey H, O'Malley L (2005). Scoping studies: towards a methodological framework. Int J Soc Res Methodol.

[25] Downing J, Powell RA, Marston J, Huwa C, Chandra L, Garchakova A, Harding R (2016). Children’s palliative care in low-and middle-income countries. Arch Dis Child.

[26] Downing J, Birtar D, Chambers L, Gelb B, Drake R, Kiman R (2012). Children’s palliative care: a global concern. Int J Palliat Nurs.

[27] Hessissen L, Madani A (2012). Pediatric oncology in Morocco: achievements and challenges. J Pediatr Hematol Oncol.

[28] Shad A, Ashraf MS, Hafeez H (2011). Development of palliative-care services in a developing country: Pakistan. J Pediatr Hematol Oncol.

[29] Silbermann M, Arnaout M, Daher M, Nestoros S, Pitsillides B, Charalambous H, Gultekin M, Fahmi R, Mostafa K, Khleif A (2012). Palliative cancer care in middle eastern countries: accomplishments and challenges. Ann Oncol.

[30] Collins K, Harding R (2007). Improving HIV management in sub-Saharan Africa: how much palliative care is needed?. AIDS Care.

[31] Delgado E, Barfield RC, Baker JN, Hinds PS, Yang J, Nambayan A, Quintana Y, Kane JR (2010). Availability of palliative care services for children with cancer in economically diverse regions of the world. Eur J Cancer.

[32] Cardenas-Turanzas M, Tovalin-Ahumada H, Romo CG, Okhuysen-Cawley R (2015). Assessing need for palliative Care Services for Children in Mexico. J Palliat Med.

[33] Nakawesi J, Kasirye I, Kavuma D, Muziru B, Businge A, Naluwooza J, Kabunga G, Karamagi Y, Akankwasa E, Odiit M: Palliative care needs of HIV exposed and infected children admitted to the inpatient paediatric unit in Uganda. ecancermedicalscience 2014, 8, 489. 10.3332/ecancer.2014.489 PMC4303617 25624870

[34] Kuan GL, Low W (2015). Y: Parents' perspectives on the important aspects of care in children dying from life limiting conditions: a qualitative study. Med J Malaysia.

[35] Weaver M, Yao A, Renner L, Harif M, Lam C (2015). The prioritisation of paediatrics and palliative care in cancer control plans in Africa. Br J Cancer.

[36] Amery JM, Rose CJ, Holmes J, Nguyen J, Byarugaba C: The beginnings of children's palliative care in Africa: evaluation of a children's palliative care service in Africa. J Palliat Med 2009, 12(11):1015-1021. 10.1089/jpm.2009.0125 19922001

[37] Connor SR, Sisimayi C, Downing J, King E, Ken PLA, Yates R, Marston J (2014). Assessment of the need for palliative care for children in South Africa. Int J Palliat Nurs.

[38] The World Bank. The World Bank: Data. 2014. http://data.worldbank.org. Accessed 25 March 2017.

[39] Harding R, Brits H, Penfold S: Paediatric antiretroviral therapy outcomes under HIV hospice care in South Africa. Int J Palliat Nurs 2009, 15(3). 10.12968/ijpn.2009.15.3.41093 19537535

[40] Rawlinson F, Gwyther L, Kiyange F, Luyirika E, Meiring M, Downing J: The current situation in education and training of health-care professionals across Africa to optimise the delivery of palliative care for cancer patients. ecancermedicalscience 2014, 8, 492. 10.3332/ecancer.2014.492 PMC4303614 25624873

[41] International Children's Palliative Care Network: elearning. http://www.elearnicpcn.org/. Accessed 12 May 2016.

[42] Berterame S, Erthal J, Thomas J, Fellner S, Vosse B, Clare P, Hao W, Johnson DT, Mohar A, Pavadia J (2016). Use of and barriers to access to opioid analgesics: a worldwide, regional, and national study. Lancet.

[43] NHS Choices. https://www.nhs.uk/Planners/end-of-life-care/Pages/what-it-involves-and-when-it-starts.aspx. Accessed 10 April 2017.

[44] Harding R, Albertyn R, Sherr L, Gwyther L (2014). Pediatric palliative care in sub-Saharan Africa: a systematic review of the evidence for care models, interventions, and outcomes. J Pain Symptom Manag.

