# Supplementary material for: A scoping review of palliative care for children in low- and middle-income countries
Source: BMC Palliat Care. 2017 Nov 25;16:60. doi: 10.1186/s12904-017-0242-8 (PMC5702244; doi:10.1186/s12904-017-0242-8)
Supplement: Additional file 1: — The discription of the search strategy. (DOCX 20 kb) [file 12904_2017_242_MOESM1_ESM.docx]

**Additional file 1:**

**The following search terms were used for Pubmed:**

((child[Title/Abstract] OR children[Title/Abstract]) OR adolescent[Title/Abstract]) OR adolescence[Title/Abstract]) OR infant[Title/Abstract]) OR baby[Title/Abstract]) OR youth[Title/Abstract]) OR young adult[Title/Abstract]) OR paediatrics[Title/Abstract]) AND ("palliation"[Title/Abstract] OR "palliative care"[Title/Abstract]) OR "terminal care"[Title/Abstract]) OR "bereavement"[Title/Abstract]) OR "advance care planning"[Title/Abstract]) OR "hospice care"[Title/Abstract]) OR "hospices"[Title/Abstract]) OR "end of life"[Title/Abstract]) OR ("quality"[Title/Abstract]) OR "quality/care"[Title/Abstract]) OR "evaluation"[Title/Abstract]) OR ("model"[Title/Abstract] OR "program"[Title/Abstract]) OR "framework"[Title/Abstract]) OR "implementation"[Title/Abstract])) AND ("developing countries"[Title/Abstract] OR "low income population"[Title/Abstract] ))

(In order to maximize the number of relevant articles, the words of "needs" and "accessibility" were not included as search terms.)

Additional records by hand search using terms of `pediatric palliative care’ `low and middle income countries’ were identified through Google Scholar.

Duplications and irrelevant articles were eliminated by screening.
